# Immune checkpoint regulation is critically involved in canine cutaneous histiocytoma regression

**DOI:** 10.3389/fvets.2024.1371931

**Published:** 2024-06-19

**Authors:** Benjamin Diehl, Florian Hansmann

**Affiliations:** Institute of Veterinary Pathology, Faculty of Veterinary Medicine, Leipzig University, Leipzig, Germany

**Keywords:** dog, histiocytoma, mx1, immune checkpoint, CD80, CD86, tumor regression

## Abstract

**Introduction:**

Canine cutaneous histiocytoma (CCH) is a benign tumor frequently occurring in young dogs which is derived from Langerhans cells (LC). Distinguishing features of this tumor are its spontaneous regression following a rapid tumor growth. Impaired control of immune checkpoints during tumor development and progression is a widespread phenomenon which may result in an absent or ineffective immune response. The interaction between the inflammatory response and the expression of immune checkpoint molecules is only partially described in this tumor type. The aim of this study was to identify immune checkpoint molecules and molecules from the interferon-mediated immune response that are involved in the regression of CCH.

**Methods:**

Forty-eight CCH derived from dogs ≤ 4 years of age were assigned to one of four groups according to the severity and distribution of lymphocyte infiltration. Using immunohistochemistry and whole-slide image scans of consecutive sections the expression of programmed death protein ligand 1 (PD-L1), CD80, CD86, Survivin, forkhead box protein 3, Ki-67, cleaved caspase-3, CD3, and mx1 were investigated. RNA *in-situ* hybridization was performed for transcripts of mx1 and interferon-γ.

**Results:**

Neoplastic cells showed an expression of PD-L1, CD80, CD86, and Survivin. The density of CD80 expressing cells was negatively correlated with regression while the density of cleaved caspase-3 positive cells increased with regression. Mx1 transcripts and protein were predominantly localized in neoplastic cells while interferon-γ transcripts were most frequently detected in T-cells.

**Conclusion:**

The expression of the immune checkpoint molecules CD86 and PD-L1 and particularly the reduced expression of CD80 in groups 3 and 4 indicate an influence of the investigated immune checkpoints on tumor regression. In parallel an activation of the apoptotic cascade during regression is suggested. Finally, the detection of mx1 within the neoplasm pinpoints to a yet undisclosed role of anti-cellular signaling in tumor immunity.

## 1 Introduction

Canine cutaneous histiocytoma (CCH) is a benign neoplasm of young dogs, originating from Langerhans cells (LC). These tumors show a rapid growth associated with a high proliferation rate of neoplastic cells. Interestingly, spontaneous regression occurs after some time (1). Studies investigating this entity have noted a change in phenotype and cytomorphology during CCH regression and attributed the increased immune cell infiltration to a more mature phenotype of the tumor cells (2–5). In addition, the ability of CCH derived tumor cells to stimulate lymphocytes was shown *in vitro* (6). During regression of CCH the infiltration of lymphocytes follows a characteristic pattern, which is used for division into four tumor groups (7). The predominant lymphocyte population in groups two through four consists of cluster of differentiation (CD)8 positive lymphocytes while in group one a mild prominence of CD4 positive lymphocytes is present (8). Further investigation into cytokines revealed a pro-inflammatory mix consisting of TNF-α, interleukin (IL)-2 and interferon-γ (IFN-γ) (8). No etiology or trigger for either tumorigenesis or initiation of tumor regression has been identified yet.

While tumor regression is defined by a reduction of tumor cells in number, a high rate of apoptosis does not necessarily correlate with tumor regression and may also occur in growing neoplasms. Important factors responsible for a reduced tumor growth are immune-mediated (i.e., cytotoxic) events, terminal differentiation and senescence (9, 10). The tumor microenvironment is extensively modulated by the neoplastic cells. One mechanism, by which the tumor cells escape the host’s immune response is the hijacking of immune-checkpoints, like programmed cell death ligand 1 (PD-L1), CD80, CD86 and their respective receptors, programmed death receptor 1 (PD-1) and cytotoxic T-cells associated protein 4 (CTLA-4). PD-L1 is able to suppress the cytotoxic immune response by multiple mechanisms including the induction of T-cell apoptosis and exhaustion as well as the stimulation of regulatory T-cells (11, 12). While CD80 and CD86 can deliver a co-stimulatory signal by the CD28 receptor, the suppressive signals are mediated by binding to CTLA-4, exhibiting a greater affinity. CD28 is expressed on virtually all T-cells, while CTLA-4 is limited to activated and regulatory T-cells (13). The competitive binding as well as secondary signaling result in an attenuation of T-cell activity and apoptosis. The expression of these molecules as well as their possible therapeutic potential have been described in some types of canine cancer (14–18).

IFN-γ is the sole type-II-interferon with an ambiguous role in cancer (19). On the one hand it has an established role in T-cell activation, dendritic cell maturation and programming of tumor-associated macrophages into an anti-tumor phenotype but on the other hand it plays a role in natural killer cell suppression (20, 21). MX Dynamin GTPase 1 (mx1), one of the IFN-stimulated genes, is mainly involved in the antiviral immune response and is induced by type-I-interferons (22). While the exact mechanisms remain not fully understood, it has been described inhibiting viral transcription and replication (22–24). Furthermore, mx1 has also been described in human cancer, with the presence of mx1 being linked to metastatic potential in two studies on colorectal and breast cancer (25, 26).

The aim of this study was to investigate the contribution of immune checkpoint molecules to the regression of CCH. The hypothesis was that group specific differences in cell-survival, e.g., Survivin, mitotic count and Ki-67, as well as apoptosis (cleaved caspase-3) in association with the expression of specific immune checkpoint molecules exist. Since the regression of CCH is associated with a group-dependent increasing number of lymphocytes, pro-inflammatory molecules including IFN-γ and mx1 were investigated and assigned to their predominantly expressing cell population.

## 2 Materials and methods

### 2.1 Characterization of samples

For this study 48 formalin fixed paraffin embedded (FFPE) skin samples diagnosed with CCH from dogs ≤ 4 years of age were selected from the archive of the Institute of Veterinary Pathology, Leipzig University. All specimens were screened for the extent of secondary inflammation and classified into four groups according to the degree and distribution of infiltrating lymphocytes as previously described (7). The study design comprised four groups in which CCH of 12 animals per group (6 female and 6 male) were examined. The diagnosis of CCH was confirmed via immunohistochemistry using ionized calcium-binding adapter molecule (Iba)-1 and exclusion of other round cell neoplasms like histiocytic sarcoma (CD204), cutaneous T-cell lymphoma (CD3) and mast cell tumor (CD117) antibodies in addition to hematoxylin–eosin (H&E) stain. Further, mitotic count in tumor cells was evaluated per mm^2^ by counting mitotic figures in 10 consecutive and randomly chosen areas of 0.237 mm^2^ (2.37 mm^2^ in total) using a microscope at 400-fold magnification and a field number of 22.

### 2.2 Immunohistochemistry

Antibodies used for immunophenotyping are summarized in Table 1. Positive control tissues known to include the target epitope were used as indicated in Table 1. For negative controls the primary antibody was replaced by normal serum from the respective species of the primary antibody in an equivalent concentration as the primary antibody on CCH slides as well as positive control tissues. Consecutive sections were mounted on positively charged glass slides (New Erie Scientific LLC, Portsmouth, NH, United States) followed by deparaffinization and rehydration. Afterwards endogenous peroxidase was inactivated with methanol and 0.5% hydrogen peroxide. Epitope retrieval was performed by incubation in either ethylenediaminetetraacetic acid (EDTA; pH 9.0) or citrate buffer (pH 6.0) at 96°C for 25 min in a water bath. For CD3 a protease-based epitope retrieval using 0.5 g/L protease (derived from *Bacillus licheniformis,* Sigma-Aldrich, St. Louis, MO, United States) in phosphate buffered saline for 5 min at 37°C was applied. Goat serum (Biozol Diagnostica Vertrieb GmbH, Eching, Germany) at a dilution of 5% was used for 30 min in order to block unspecific antibody binding. This was followed by incubation with the primary antibody. After incubation at 4°C overnight, secondary antibodies were incubated at room temperature for 30 min. For visualization, avidin-biotin-complex (ABC; Vector Laboratories, Newark, CA, United States) with 3,3’-diaminobenzidine (DAB; Sigma-Aldrich, St. Louis, MO, United States) and Mayer’s hematoxylin counterstain was used in all cases.

**Table 1:** Antibodies used in the study.

Target molecule	Control tissue	Supplier/Product number	Working concentration	Epitope retrieval	Host/Clonality	Secondary antibody	Supplier/Product number	Detection system	References
Cleaved caspase-3	Tonsil	R&D systems, AF835	0.2 μg/mL	citrate	Rabbit/polyclonal	Goat-anti-rabbit	Vector LaboratoriesBA-1000	ABC	Not applicable
CD3	Tonsil	Dako, A0452	2 μg/mL	protease	Rabbit/polyclonal	Goat-anti-rabbit	Vector LaboratoriesBA-1000	Envision	Not applicable
CD80	Tonsil	Bioss, Bs-10340R	2.5 μg/mL	citrate	Rabbit/polyclonal	Goat-anti-rabbit	Vector LaboratoriesBA-1000	ABC	Antibody targeting KLH conjugated synthetic peptide derived from canine CD80/B7-1 according to manufacturer
CD86	Tonsil	Antibodies-Online, ABIN736701	1.25 μg/mL	citrate	Rabbit/polyclonal	Goat-anti-rabbit	Vector LaboratoriesBA-1000	ABC	(27)
CD117	Mast cell tumor	Dako, A4502	12.6 μg/mL	citrate	Rabbit/polyclonal	Goat-anti-rabbit	Vector LaboratoriesBA-1000	ABC	Not applicable
CD204	Histiocytic sarcoma	Trans genic Inc., SRA-E6	0.5 μg/mL	citrate	Mouse/monoclonal	Goat-anti-mouse	Vector LaboratoriesBA-9200	ABC	(28)
FoxP3	Tonsil	eBioscience, FJK-16 s	10 μg/mL	citrate	Rat/monoclonal	Rabbit-anti-rat	Vector LaboratoriesBA-4000	ABC	(29)
Iba-1	Lymph node	Novus bio, NBP2-19019	0.06 μg/mL	citrate	Rabbit/polyclonal	Goat-anti-rabbit	Vector LaboratoriesBA-1000	ABC	Not applicable
Ki67	Small intestine	Dako, MIB-1 M7240	0.92 μg/mL	citrate	Mouse/monoclonal	Goat-anti-mouse	Vector LaboratoriesBA-9200	ABC	Not applicable
Mx1	Brain (canine distemper infected)	M143 (provided by Prof. Kochs, Institute of Virology, Freiburg University)	0.6 μg/mL	citrate	Mouse/monoclonal	Goat-anti-mouse	Vector LaboratoriesBA-9200	ABC	(23, 30, 31)
PD-L1	Tonsil	Abcam, 233482	0.5 μg/mL	EDTA	Rabbit/polyclonal	Goat-anti-rabbit	Vector LaboratoriesBA-1000	ABC	Recombinant fragment (His-tag) corresponding to Human PD-L1 aa 1–250 (81.06% protein homology with canine PD-L1)
Survivin	Squamous cell carcinoma	Novus bio, NB500-201	5 μg/mL	citrate	Rabbit/polyclonal	Goat-anti-rabbit	Vector LaboratoriesBA-1000	Envision	Antibody against full length recombinant humanSurvivin [UniProt# O15392](32, 33)

All antibodies were established on canine tissue using dilution series and different pretreatments. Immunoreactive cells were checked for morphology, localization and distribution within the positive control tissue as well as the target tissue. Where relevant, previous references reporting antibody validation for use in canine tissues or the source of the immunogen sequence used for generating the primary antibody including sequence homology to the respective canine sequence were indicated. ABC, avidin-biotin-complex; CD, cluster of differentiation; EDTA, ethylenediaminetetraacetic acid; FoxP3, forkhead box protein 3; Iba-1, ionized calcium-binding adapter molecule 1; KLH, keyhole limpet hemocyanin; Mx1, myxovirus resistance protein 1; PD-L1, programmed death ligand 1.

### 2.3 Quantitative analysis using QuPath

Whole slide images (WSI) were obtained using an Axioscan 7.0 with Plan-Apochromat 20x/0.8 lens (200x magnification; Zeiss Group, Jena, Germany). Subsequently, WSI were subjected to image analysis using QuPath (version 0.4.2) (34). Staining parameters were manually set by choosing representative regions for hematoxylin and DAB staining. Tumor area for each slide was selected manually as region of interest (ROI). For each slide 10 areas with 500 × 500 μm (equals 2,5 mm^2^ in total per slide) were created with QuPath’s “Tiles” function. The number of positive cells per mm^2^ were then determined in representative areas, with a manually chosen threshold applied to evaluate the DAB signal in either the nuclear or cytoplasmic cell compartment. Cytoplasmic staining was considered for CD3, Iba-1, Survivin, CD80, CD86 and cleaved caspase-3 and nuclear staining for forkhead box protein 3 (FoxP3) and Ki-67. Both the number of positive cells per mm^2^ and the percentage of positive cells in relation to the total number of cells were evaluated. Only tumor cells were evaluated for CD80, CD86, PD-L1, Survivin and Ki-67. A minimum of 5% of immunolabeled tumor cells per case were used as a cut-off value for CD80, CD86, PD-L1 and Survivin. The analysis of Iba-1 and CD3 positive cells was performed using a whole-slide approach including the entire tumor area.

For the evaluation of Ki-67, PD-L1, CD80, CD86, Survivin and FoxP3 immunohistochemistry an algorithm using QuPath’s “Train classifier” tool with “random tree” method to differentiate immune and tumor cells was trained using selected WSI of all CCH groups. Single or few cells were manually annotated as either “immune” or “tumor” identity until the classifier resulted in satisfactory classification in all ROIs. Main discriminatory factors in choosing the phenotype were size, shape, color as well as amount and texture of visible cytoplasm. After this step was repeated for all training slides, the results were visually validated in representative areas of randomly chosen slides. This involved the counting of 100 cells and a cross-check of their phenotype against the classification provided by the algorithm. The resulting classifier was subsequently run on the previously selected 500 × 500 μm fields to exclude immune cells for Ki-67, PD-L1, CD80, CD86 and Survivin and tumor cells for FoxP3 evaluation, respectively. As a last step of validation, the percentage of “immune” cells recognized by the algorithm per group was checked for similarity with the percentage of CD3-immunolabeled cells and examined for changes between the groups.

### 2.4 *In-situ* hybridization

*In-situ* hybridization was used to visualize and quantify IFN-γ and mx1 transcripts. Using a biopsy punch with 3 mm diameter, one core of each neoplasm (n = 48) with a representative cell composition was taken. Cores were randomly assigned to one of four blocks consisting of 12 cores each. The RNAscope technology was used for the identification of IFN-γ and mx1 (Advanced Cell Diagnostics, Inc., Minneapolis, MI, United States) according to the manufacturers’ instructions. The probes were designed using a canine sequence for IFN-γ (Entrez Gene ID 4038014) and mx1 (Entrez Gene ID 403745) targeting IFN-γ and mx1, respectively. For mx1 inflamed brain tissue from a canine distemper encephalitis case served as positive control (30). Since IFN-γ expression in CCH is previously described (8) no additional positive control tissue was included. Process controls consist of a negative control (RNAscope® Negative Control Probe—dapB) and a positive control (RNAscope® Probe—*Canis lupus familiaris* peptidylprolyl isomerase B) according to manufacturers’ instructions. Negative controls were checked for the absence of specific labeling. For identification of the source of IFN-γ and mx1 expression sequential serial slides were stained for CD3 and Iba-1 using immunohistochemistry. Slides were digitalized using the Axioscan’s 40×/0.65 lens.

The quantification of IFN-γ and mx1 transcripts was undertaken as follows: Firstly, positive pixels within the area of each individual core were measured using a pixel-based classifier (“positive pixel”) and expressed in percentage. Next, the number of positive cells with at least 1% positive pixels (“positive cells”) was identified and followed by execution of the cell detection function of QuPath. This resulted in the number of positive pixels per cell (“positive signal”) as a measure of the intensity of the labeling. From all measurements of all cells of one core a median value was calculated as indicator for the positive signal per positive cell per sample. Median values served as variable for the comparison between the investigated neoplasms.

Lastly, to determine the cell’s phenotype, the Interactive Image Alignment tool (v0.3.0) was used, allowing an overlay of two or multiple slides within QuPath. Whenever possible, 50 cells positive for mx1 or IFN-γ signal were manually selected for each individual core. Next, consecutive slides immunostained for CD3 and Iba-1 were used as overlay and cells were labeled according to the immunophenotype as “iba-1” or “cd3,” respectively. If the cell could be identified in both immunohistochemistry slides and displayed neither signal, they were labeled as “other.” Cells that were not consistently shown in all three slides or only shown in one immunohistochemical slide as immunonegative were labeled as “mismatch.” Generally, it was assumed that individual cells either expressing Iba-1 or CD3 do not express the other marker. Overall, for IFN-γ only 16 cores could be evaluated due to difficulties in overlay, resulting in 800 evaluated cells. For mx1 44 cores were suitable for evaluation with 2,179 evaluated cells.

### 2.5 Statistical analysis

Statistical analysis was made with R 4.2.1 using RStudio and the *tidyverse* package (35–37). Specimens were grouped according to group and measurements checked for normal distribution using Q-Q-plots. Subsequently, medians and quantiles were calculated and statistical significance between tumor group and sex was evaluated using the Kruskal-Wallis test and Wilcoxon-rank-sum test for *post hoc* analysis. For the analysis of the *in-situ* hybridization the median of all cells per core were evaluated. A *p*-value < 0.05 was chosen as threshold for statistical significance. Possible correlation was analyzed using Spearman’s ρ.

## 3 Results

### 3.1 Characterization of CCH

**Figure 1:**
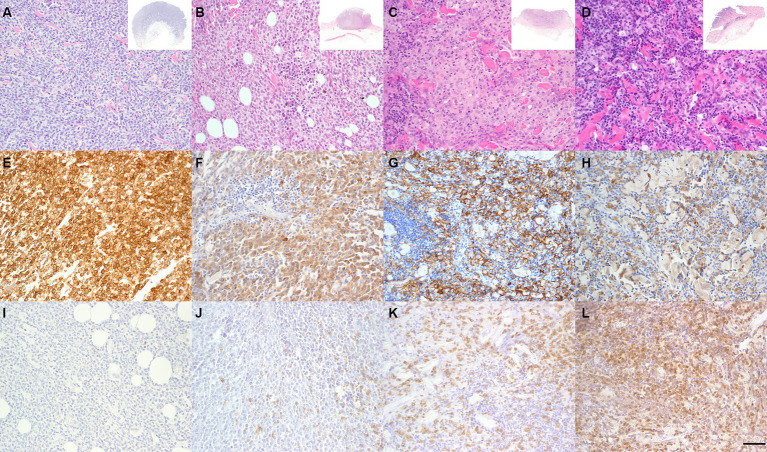
Characterization of group-dependent changes in canine cutaneous histiocytoma (CCH). CCH showed a group-dependent decrease of tumor cells in parallel with an increase of infiltrating lymphocytes. Group 1–4 **(A–D)** with overview shown in inset. Hematoxylin–Eosin (HE). Tumor cells showed a strong, group-independent immunolabeling for ionized calcium-binding adapter molecule 1 (Iba-1) with a decline of Iba-1 labeled cells between groups **(E–H)**. Immunophenotyping of inflammatory cells revealed a group-dependent increasing number of CD3 positive T-cells (**I–L**, bar 100 μm).

The study included 48 CCH with 12 samples each belonging to groups 1 to 4, respectively (Figure 1). No significant differences were observed between the epitopes investigated with regard to sex or breed. Consequently, specimens were evaluated solely in relation to their assigned group. Mean age at excision was 1.84 years. Neoplastic cells were positive for Iba-1 and negative for CD204, CD117 and CD3.

Iba-1 positive cells in tumor ROI showed a significant decline between groups from 3288.8 cells per mm^2^ (85.26%) to 2523.3 per mm^2^ (53.22%), while CD3 positive cells showed an increase from 1039.9 per mm^2^ (11.3%) to 3225.4 per mm^2^ (53.5%) in group 1 and 4, respectively (*p* < 0.05; Figure 1). Mitotic count in tumor cells differed between groups with group 1 and 2 (9.3 and 9.5 mitotic figures per mm^2^, respectively) having significantly higher counts than groups 3 and 4 (4.0 and 0.6 per mm^2^, respectively; *p* < 0.05). Overall, there was a high heterogeneity of the mitotic count between samples with values varying between 0 and 21.1 per mm^2^ and an overall median of 11.5 mitoses per mm^2^.

### 3.2 Immune checkpoint expression

**Figure 2:**
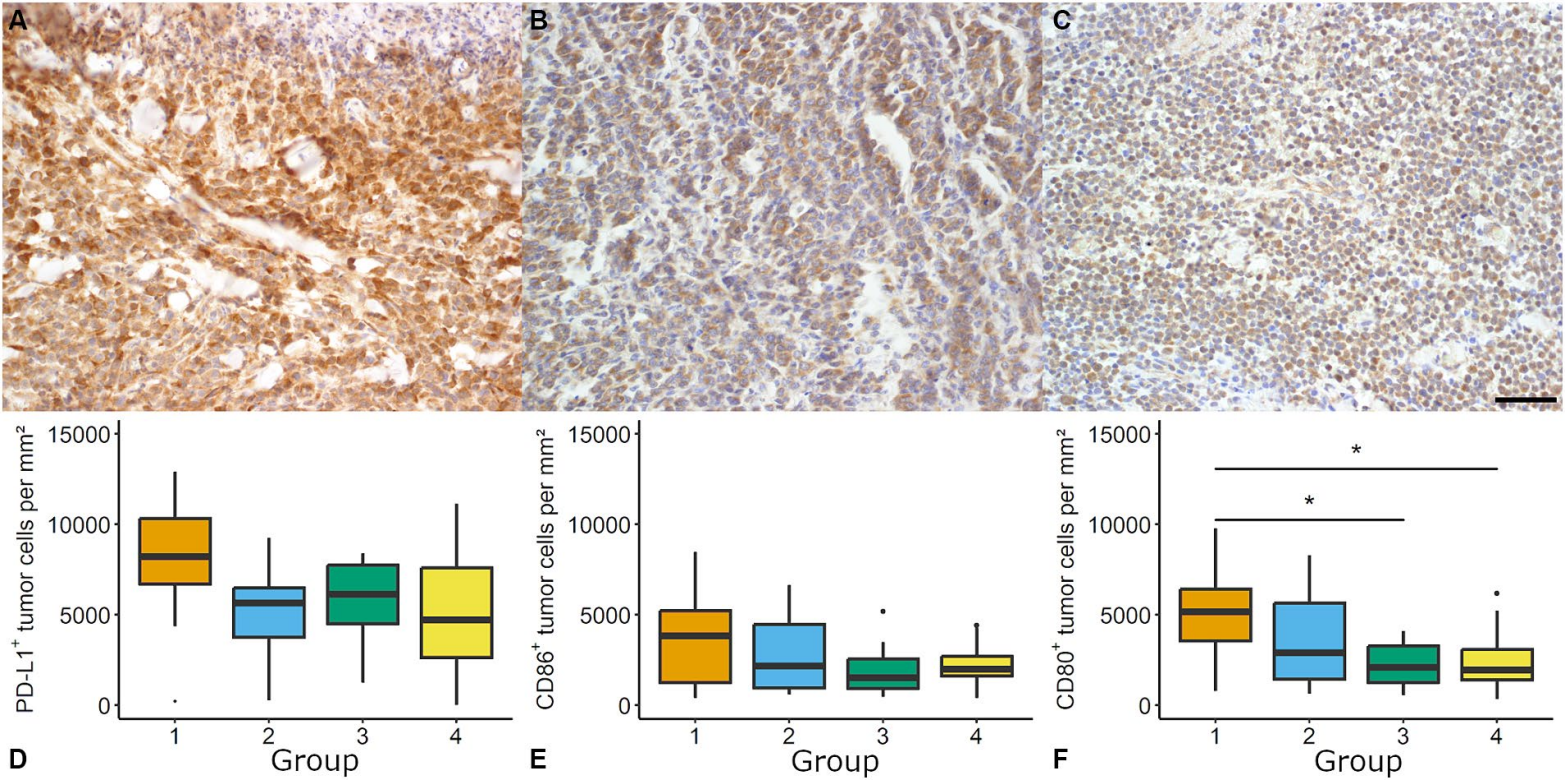
Evaluation of immune checkpoints in canine cutaneous histiocytoma (CCH). CCH revealed a high density of programmed death ligand 1 (PD-L1) immunolabeled cells (**A**, group 2) and moderate densities of CD86 (**B**, group 1) and CD80 (**C**, group 1, bar = 100 μm) immunopositive cells. For PD-L1 **(D)** and CD86 **(E)** no group-dependent effect was observed. The density of CD80 positive cells showed a group-dependent decrease (Kruskal-Wallis test followed by Wilcoxon-rank-sum test, asterisk = *p* < 0.05; **F**). Boxplots show median (line) and interquartile range (box).

Immune checkpoint evaluation revealed an expression of CD80 and PD-L1 in all samples (48/48), while 45/48 showed considerable levels of CD86 (Figure 2).

PD-L1 immunolabeling showed a variable degree of staining intensity. Samples showed a group-independent high number of PD-L1 immunolabeled tumor cells (overall median: 8748.0 cells per mm^2^ and 73.89%, respectively).

The density of CD80 immunolabeled tumor cells showed a significant decline from group 1 to groups 3 and 4 (e.g., 5161.6 positive tumor cells per mm^2^ in group 1 and 1941.12 positive tumor cells per mm^2^ in group 4; *p* < 0.05; Figure 2). Significant differences between the groups with regard to percentage values were identified. These differences were detected between group 1 (43.44%) and group 3 (22.38%) but not between group 1 and group 4 (29.05%). The density of CD86 immunolabeled tumor cells showed no group-related differences. Median expression ranged between 1519.93 positive tumor cells per mm^2^ (21.69%; group 3) and 3846.21 positive tumor cell per mm^2^ (30.28%; group 1).

No correlation was found for CD3 and CD80, CD86 or PD-L1 positive (tumor) cells per mm^2^, respectively. A moderate correlation was found for mitotic count and CD80 (Spearman’s ρ = 0.49, *p* < 0.05) as well as mitotic count and CD86 positive tumor cells per mm^2^ (ρ = 0.34, *p* < 0.05).

### 3.3 Markers for cell proliferation and apoptosis

**Figure 3:**
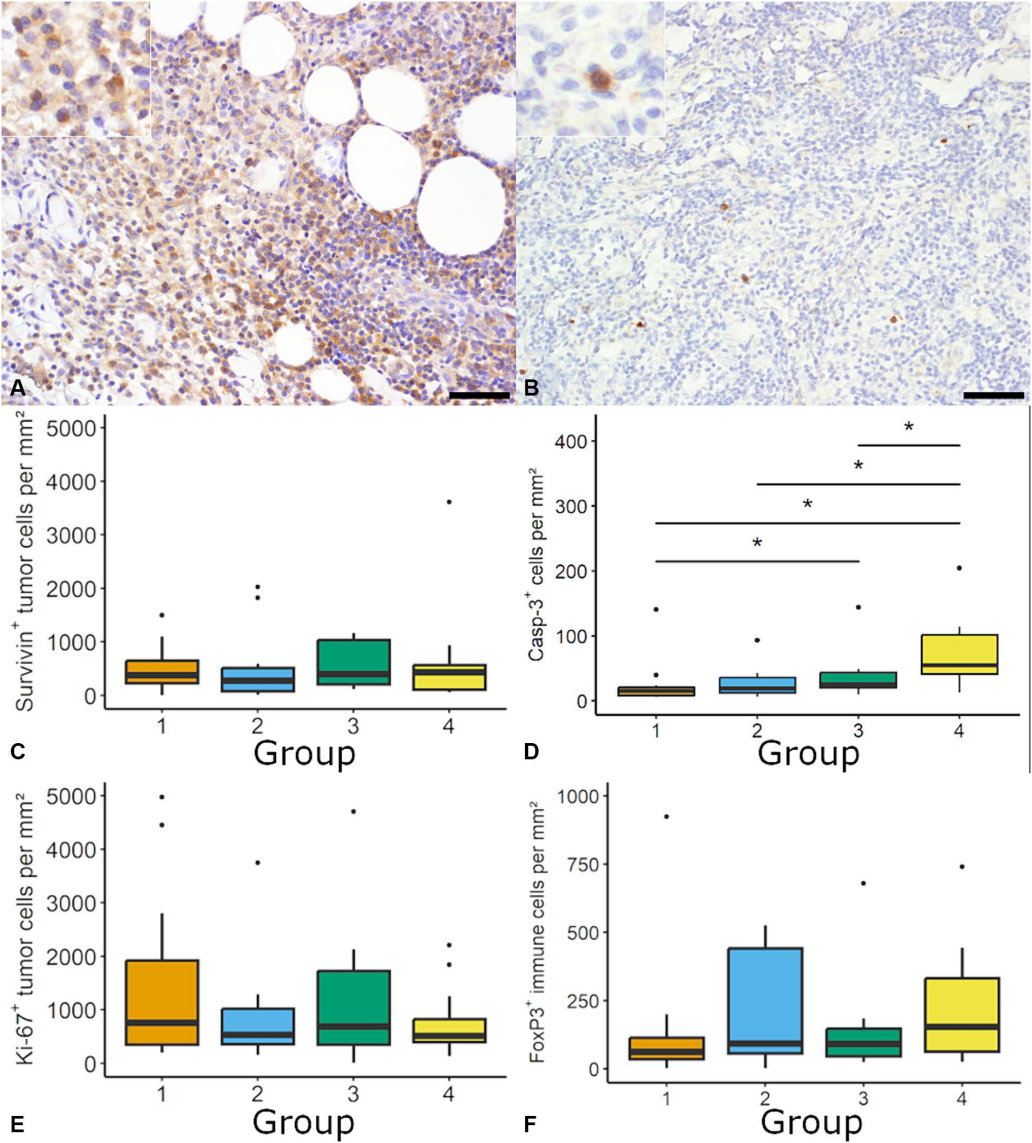
Quantification of cell proliferation, apoptosis and regulatory T-cells in canine cutaneous histiocytoma (CCH). Survivin was most frequently expressed within the cytoplasm (**A**; group 2; inset: higher magnification) and cleaved caspase-3 (Casp-3) positive cells showed a diffuse cytoplasmic labeling (**B**; group 3; inset: higher magnification). The density of Survivin immunolabeled cells was stable throughout groups **(C)**. A group-dependent increase of the density of Casp-3 positive cells (Kruskal-Wallis-Test followed by Wilcoxon-rank-sum test, asterisk = *p* < 0.05) was observed **(D)**. The density of Ki-67 positive tumor cells and regulatory T-cells (forkhead box protein 3; FoxP3) was similar among tumor groups **(E,F)**. Boxplots show median (line) and interquartile range (box).

Regarding cell proliferation, the mitotic count was supplemented by the investigation of Ki-67 and Survivin (Figure 3). For Ki-67 the median number ranged between 521.4 tumor cells per mm^2^ (6.56%) in group 2 and 745.4 tumor cells per mm^2^ (11.55%) in group 1 with no significant differences between groups. The distribution of Ki-67 positive tumor cells was heterogenous within and between samples. Individual samples showed variable numbers of Ki-67 positive tumor cells ranging between 0.4 and 4966.8 tumor cells per mm^2^ (Figure 3).

The density of tumor cells with cytoplasmic Survivin expression ranged between 273.73 tumor cells per mm^2^ (3.4%; group 2) and 431.69 tumor cells per mm^2^ (6.2%; group 4) with no difference between groups and an overall median of 386.28 positive tumor cell per mm^2^ (5.1%; Figure 3). Across all groups 50% of samples showed more than 5% immunolabeled tumor cells, with no differences between groups. Nuclear expression of Survivin was occasionally observed in neoplastic cells (data now shown). A moderate positive correlation was found for positive cell per mm^2^ between Ki-67 and Survivin (ρ = 0.49; *p* < 0.05) as well as Ki-67 and PD-L1 (ρ = 0.38, *p* < 0.05).

The rate of apoptosis was determined by assessment of cells positive for cleaved caspase-3. The density of cleaved caspase-3 positive cells was significantly higher in group 4 (55.0 positive cells per mm^2^; 0.55%) compared to the other groups (e.g., group 1: 15.2 positive cells per mm^2^; 0.12%; Figure 3). A moderate negative correlation was found between the mitotic count and cleaved caspase-3 positive cells per mm^2^ (ρ = −0.47, *p* < 0.05).

### 3.4 The anti-tumor response in CCH

Lastly FoxP3, as marker for regulatory T-cells, was quantified. FoxP3 positive cells were randomly distributed with a median between 62.58 per mm^2^ (1.78%) in group 1 and 154.55 per mm^2^ (2.60%) in group 4, without significant differences between groups (Figure 3). Overall median was 94.6 cells per mm^2^ (1.86%).

**Figure 4:**
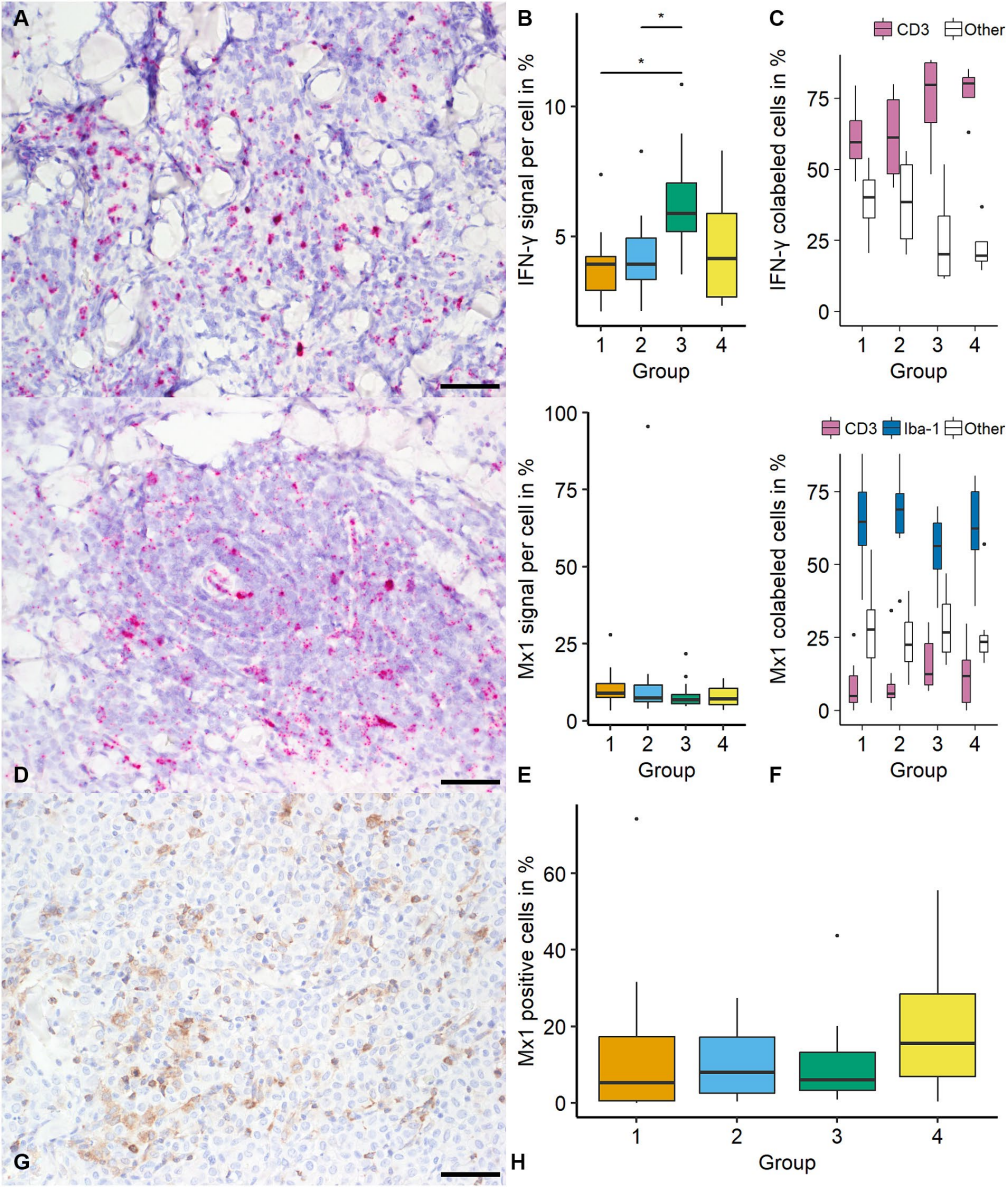
Quantification of Interferon-γ (IFN-γ) and MX Dynamin GTPase 1 (mx1) in canine cutaneous histiocytomas (CCH). IFN-γ transcripts were most frequently detected in cells showing a lymphocytic phenotype as determined using light microscopy (**A**; group 2; bar = 50 μm). An increase of IFN-γ signal (percentage positive pixel per cell) was detected between groups 1 and 2 and group 3 (**B**, Kruskal-Wallis-Test followed by Wilcoxon-rank-sum test, asterisk = p < 0.05). IFN-γ was most frequently colocalized with CD3 **(C)**. Mx1 transcripts were detected within tumor cells with no group-specific difference regarding the signal (percentage positive pixel per cell; Kruskal-Wallis-test; **D**; group 2, **E**). Mx1 was most frequently detected in ionized calcium-binding protein 1 (Iba-1) immunolabeled cells **(F)**. Mx1 expression was confirmed by immunohistochemistry (**G**; group 3) with similar results as with *in-situ* hybridization **(H)**. Boxplots show median (line) and interquartile range (box).

The percentage of IFN-γ positive pixel (with 0.66% and 1.12% positive pixels within the ROI, respectively) increased from group 1 to 4 (*p* < 0.05). In addition, the number of positive cells was higher in group 3 (34.43%) and 4 (21.08%) compared with group 1 (11.26%; *p* < 0.05). The median positive signal increased from groups 1 and 2 to group 3 (3.94%, 3.93%, and 5.89% positive pixels per cell, respectively; *p* < 0.05; Figure 4). IFN-γ was most frequently detected in CD3 immunolabeled cells (68.28%; 409/599 cells), while no tumor cells were positive for IFN-γ. A high number of IFN-γ positive cells were classified as “other” (31.72%; 190/599). There was no difference in IFN-γ expressing cells’ identity between groups (Figure 4).

For mx1 there was no difference in the percentage of positive pixels or the number of positive cells (overall median of 1.88% positive pixel and 15.21%, respectively). For mx1 positive signal showed no statistically significant difference between groups (overall median of 7.57% positive pixels per cell; Figure 4). Mx1 was most frequently colocalized with Iba-1 (63.75%; 925/1,451 cells) with lower frequencies of CD3 positive cells (10.54%; 153/1,451) and “others” (25.71% 373/1,451). The identity of mx1 expressing cells showed no significant variation among CCH groups (Figure 4). The comparison of mx1 on transcript and protein level with respect to distribution and frequency showed similar results with an overall median of 7.85% immunolabeled cells (788.8 positive cells per mm^2^; Figure 4).

## 4 Discussion

The aim of the present study was to investigate the contribution of immune checkpoint regulation to the regression of CCH. Furthermore, the role of two important molecules of the IFN-cascade in CCH regression was investigated. Besides an expression of the markers CD80, CD86 and PD-L1 a group-dependent decline of CD80 was detected. The expression of mx1 is a novel finding not previously reported in canine neoplasia.

When discussing the importance of immune checkpoints in CCH it is important to consider that LC are antigen-presenting cells (APC) and expression of CD80 but not CD86 in LC has been described in healthy canine skin (38). While both can serve as costimulatory molecules by binding to CD28, it has been suggested that the activation of CD80 is involved in the induction of a primary immunotolerance by binding to CTLA-4 (39). Therefore, the decline of CD80 positive cells in the present study may point to the activation of T-cell mediated anti-tumor immune response and a difference between the function of CD86 and CD80 in CCH seems likely. The correlation of CD86 and CD80 with clinical outcome in canine histiocytic disease has not been reported so far, however the expression of their receptor CTLA-4 on tumor cells is associated with a worse prognosis in other malignant tumors in dogs (16, 40, 41).

Interestingly, both the group-independent expression of CD86 and the group-dependent decline of CD80 immunolabeled cells are correlated with the mitotic count. Although the present data does not allow a mechanistic insight, this may point to an influence of the tumor microenvironment on tumor proliferation.

The constitutive expression of CD86 by LC in the present study represents a novel finding. Lesions in human LC histiocytosis (LCH) occurring in the skin are characterized by an expression of CD86, CD80 and membranous major histocompatibility complex II (MHC-II), indicating a mature phenotype (42). Lesions not confined to the skin mostly lack expression of CD86 but show expression of markers associated with immaturity (e.g., CD14, CD68 and intracellular MHC-II; 42). Spontaneous regression of dermal human LCH (Hashimoto-Pritzker-disease) is dependent upon the maturity of the involved LC and their ability to trigger an immune response (42). Thus, the observed expression of CD86 and CD80 in a spontaneously regressing LC lesion in canines may highlight a translational aspect between human and canine histiocytic disease. The observed correlation of CD80 with tumor regression warrants further research into possible anti-tumor strategies in malignant histiocytic neoplasia.

PD-L1 is expressed in a variety of human histiocytic neoplasms, including LCH, but not in normal human LC (43–45). Further, its expression has not been demonstrated in canine histiocytic sarcoma, but in canine lymphoma (18, 43, 46, 47). Monoclonal antibodies blocking the PD-1:PD-L1 axis have successfully been used for the treatment of cancer in humans while the use of these antibodies in dogs is partially effective (48). This underlines the potential therapeutic significance of PD-L1 expression by different canine neoplasia. Although an expression of PD-L1 and correlation with the mitotic count could be shown, the mechanisms of PD-L1 in CCH remain elusive.

Survivin belongs to the family of inhibitor of apoptosis molecules and plays an essential role in mitosis as part of the chromosomal passenger complex. Due to its dual role in promoting cell proliferation and its ability to inhibit apoptosis, overexpression of Survivin likely promotes the growth of neoplasia. Survivin is frequently overexpressed in various neoplasia (49). The dominance of intracytoplasmic localization is in accordance with studies describing its biological function in anti-apoptosis (50). In a study investigating the expression via RT-PCR in histiocytic sarcoma cells, Survivin has been linked to a more aggressive behavior (32). Although an expression of Survivin has been detected in CCH, its effect upon tumor growth and subsequent regression remains unclear. In the present study, a moderate correlation between the density of Ki-67 and Survivin-immunolabeled cells was detected suggesting the involvement of the pro-mitotic function of Survivin in the cell cycle regulation in CCH.

In agreement with previous studies, a declining proliferation of tumor cells was detected using the mitotic count (29), but not by using Ki-67 scoring (51). In the tumor cells, mitotic count did moderately correlate with Ki-67. The decline should be interpreted with some caution as there is a possible bias due to the decrease of tumor cells per mm^2^ in later stages and a higher number of lymphocytes (29).

While earlier studies have investigated the apoptotic rate using the terminal deoxynucleotide transferase mediated dUTP nick end labeling (TUNEL) assay, we used immunohistochemistry for cleaved caspase-3 (8, 51). Caspase-3 is part of the apoptotic cascade and its activation leads to the induction of cell death. In the present study, an increased density of cleaved caspase-3 positive cells was detected during tumor regression while Ki-67 expression remained stable.

The immunomodulatory effects of FoxP3 cells in cancer include suppression of the immune response. This is shown in a study in melanocytic neoplasia in dogs which revealed cut-off values as low as 6.9 cells per HPF to be associated with tumor related death (41). The median number of FoxP3 positive cells per mm^2^ were markedly higher in the present study and ranged between 62.58 and 91.37 positive cells per mm^2^ with an overall median of 94.6 positive cells per mm^2^. Interestingly, a high heterogeneity between samples, with some showing up to 20% of immune cells to be FoxP3 positive was detected. These findings are in accordance with another study investigating the number of FoxP3 cells in CCH (29). As we have not found any data supporting a direct immunomodulatory effect of FoxP3 positive cells in CCH it remains to be clarified whether these cells are associated with tumor regression.

IFN-γ is a type-II-interferon which generally elicits an anti-cellular response and has been described as fundamental for an anti-tumor response (52). In tumor immunity, its main effects include activation of cytotoxic cells (i.e., CD8^+^ cells and macrophages) and strengthening of innate immunity. As described in previous studies, IFN-γ expression increases during regression and is part of the immune response (7, 8, 29). In this study, IFN-γ transcripts were most frequently localized in CD3 positive cells and in a smaller subset of neither CD3 nor Iba-1 (“other”) positive cells. It seems likely this subset is at least partially made up by natural killer cells, as they are one of the major cell types producing IFN-γ and play an important role in cellular immunity. Additionally to its pro-inflammatory role and the sustainment of an anti-cellular immune response it plays a role in the maturation of DC (by mediating expression of MHC-II) and thus could possibly contribute to the LCs’ maturation which has been linked to tumor regression (3, 20). However, it remains unclear whether the initial trigger for regression are the tumor cells themselves or an exogenous antigen.

Mx1 is a transcription factor triggered by type-I-interferons. Studies revealed an important role in antiviral immunity and the proposed mechanisms include disturbance of RNA viral replication (22). Recently, the expression of mx1 has been reported in some human cancers and linked to their biological behavior albeit the exact role remains undetermined (22, 25, 26).

We were able to show an expression of mx1, which did not correlate with group. While some, mostly older, studies suggested a possible (viral) etiology of CCH, there is no conclusive evidence (7, 53–56). The expression of interferon-type-I has not been investigated in CCH, so far. However, it seems plausible that the inflammatory state of CCH is triggered by a combination of type-I and type-II-interferons as both play pivotal roles in anti-tumor immunity. The presence of mx1 is plausibly part of a yet undefined role in cancer biology.

Some limitations to this study warrant specific mentioning as they are inherent to the methods used. Firstly, a differentiation of tumor cells from tumor-associated macrophages by immunohistochemistry is not possible as available antibodies are not usable on FFPE material (1). Thus, especially in late regression the number of tumor cells might be overestimated. Further, algorithms might not be able to differentiate between cleaved caspase-3 positive tumor and immune cells, especially in late stages of apoptosis. Lastly, the effect of immune checkpoints in tumor regression and progression can only be estimated by immunohistochemistry and the level of expression needed for a biological effect is still under investigation. Nevertheless, several studies suggest that a cut-off value of 5% is sufficient to induce a biological effect (43, 57).

## 5 Conclusion

In the present study, while CD86 and PD-L1 were expressed at constant levels throughout tumor growth and regression phases, the level of CD80 positive cells was shown to decline during the latter pointing to a CD80 mediated effect on tumor immune evasion in CCH. Further, an increase of cleaved caspase-3 immunolabeled cells was seen albeit no trigger or correlation could be found. The activation of the apoptotic cascade likely contributes to tumor regression. The constitutive presence of mx1 (transcripts and protein) in neoplastic cells was an unexpected finding as it has not been described in the context of canine neoplasia before. Whether and to what extent mx1 plays a role in the interaction between neoplastic cells and the pro-inflammatory immune response remains to be determined in further studies.

## Data availability statement

The raw data supporting the conclusions of this article will be made available by the authors, without undue reservation.

## Ethics statement

Ethical approval was not required for the studies involving animals in accordance with the local legislation and institutional requirements because samples were sent to the Institute of Veterinary-Pathology for diagnostic purposes. For the present study exclusively archived material was used. No samples were taken for the purpose of this study. Written informed consent was not obtained from the owners for the participation of their animals in this study because by submitting the samples to the Institute the owners confirmed that sample material can be used for research in general. However, the samples used in this study were retrospectively selected and not prospectively collected.

## Author contributions

BD: Data curation, Formal analysis, Investigation, Writing – original draft. FH: Conceptualization, Methodology, Resources, Validation, Writing – review & editing.
